# Nursing Student's Knowledge of and Attitudes Towards Pain Management: A Cross‐Sectional Study

**DOI:** 10.1002/nop2.70880

**Published:** 2026-10-15

**Authors:** Fawaz Abdullah Alshehri, Tracy Levett‐Jones, Jacqui Pich, Abdulrahman S. Alamri

**Affiliations:** ^1^ School of Nursing University of Tabuk Tabuk Saudi Arabia; ^2^ School of Nursing and Midwifery University of Technology Sydney Sydney Australia; ^3^ Department of Mathematics and Statistics, College of Science University of Jeddah Jeddah Saudi Arabia

**Keywords:** attitude, knowledge, nursing, pain management, Saudi Arabia, students, undergraduate

## Abstract

**Aim:**

The aim of this study was to examine Saudi Arabian nursing students' level of pain management knowledge and attitudes.

**Design:**

A cross‐sectional design was used in this study.

**Methods:**

The ‘Knowledge and Attitudes Survey Regarding Pain’ tool was used to measure students' knowledge of and attitudes towards pain management. Data were collected from four universities between November 2023 and January 2024 and analysed using descriptive statistics.

**Results:**

A total of 393 online surveys were submitted. The mean ‘Knowledge and Attitudes Survey Regarding Pain’ score was 44% (SD = 8.8), indicating generally low levels of knowledge and attitudes towards pain management. There were significant differences in scores across universities.

**Conclusion:**

The results of this study suggest that many of the participants had inadequate levels of knowledge and poor attitudes towards pain management. There is a need for further research to explore the factors that impact students' pain management knowledge and attitudes.

**No Patient or Public Contribution:**

No patient or public contribution in the study.

## 1 Introduction

Pain affects millions of people worldwide and the World Health Organisation (2018) identified that effective pain management is a critical health priority for improving quality of life for individuals with chronic and acute health conditions. Acute and/or persistent pain results from injuries, illnesses, medical treatments, and surgical procedures (Finnerup et al. 2022). Untreated or poorly managed pain has a substantial impact on a person's quality of life and can result in physical, emotional and economic consequences (Mills et al. 2019). Poorly managed or untreated acute pain may also cause immunological and neurological changes and lead to chronic/persistent pain (Jayawardana et al. 2021).

Carvalho et al. (2018) assert that healthcare providers are ethically responsible for alleviating pain and suffering within the current limits of knowledge and available resources. In other words, individuals who suffer from pain have the right to receive appropriate pain management (Jukić and Puljak 2018). It is important to note that the provision of effective pain management can be influenced by healthcare professionals' attitudes and knowledge levels (Miaskowski et al. 2019).

The Saudi Arabian healthcare system has undergone significant modifications with the goal of enhancing healthcare quality and accessibility (Rahman and Al‐Borie 2021). However, issues related to the effective management of pain are ongoing. This is attributed, at least in part, to nurses having an inadequate understanding of contemporary and evidence‐based pain management (Alshehri et al. 2024; Shdaifat et al. 2020). Understanding nursing students' knowledge of and attitudes towards pain management is critical for identifying educational gaps and implementing focused interventions to improve future patient care. This paper profiles a cross‐sectional study that examined Saudi Arabian nursing students' current levels of knowledge and attitudes towards pain management.

## 2 Background

In Saudi Arabian nursing programs, pain management is addressed as a component of selected nursing subjects and not as a discrete subject. It is taught using a range of modalities, including lectures, tutorials, simulations and clinical placement experiences (Maribbay et al. 2023). Previous studies have identified that Saudi nursing students often have low levels of knowledge and poor attitudes towards pain management (Alshehri et al. 2024). Further, in a cross‐sectional study conducted by Alsaqri (2018), the mean pain knowledge score of the Saudi nursing students was only 41.8% (SD = 3.71). However, the author acknowledged that due to the relatively small sample size (*n* = 150), the results could not be generalised to a broader population. Similar results were achieved in a study by Shdaifat et al. (2020) where the mean score for the knowledge and attitudes was 42.6% (SD = 9.1). It should be noted that both Alsaqri (2018) and Shdaifat et al. (2020) stated that interpretation of the English language Knowledge and Attitudes Survey Regarding Pain (KASRP) (Ferrell and McCaffery 2014) by participants whose first language was not English was a limitation that may have impacted these results. Another descriptive cross‐sectional study by Wazqar (2019) examined Saudi female nursing students' knowledge and attitudes towards cancer pain management. Out of the total sample (*n* = 135), no participants scored more than 31%. No studies of Saudi nursing students were identified that have examined the impact of educational or healthcare initiatives to improve pain management (Rahman and Al‐Borie 2021).

The current study sought to address the key limitations identified in these studies by (1) using an Arabic translated version of the Knowledge and Attitude Survey Regarding Pain tool; and (2) recruiting a larger sample of currently enrolled male and female nursing students from multiple Saudi nursing schools.

The aim of this study was to:

- Examine the Saudi nursing students' current knowledge of and attitudes towards pain management.
- Explore differences in pain management knowledge and attitudes between subgroups of Saudi nursing students.
- Examine demographic characteristics influencing Saudi nursing students' knowledge of and attitudes towards pain management.

## 3 Methods

### 3.1 Design, Sample and Setting

This study used a quantitative cross‐sectional survey design with an online self‐reported survey (The Knowledge and Attitudes Survey Regarding Pain). Participants included 3rd and 4th year nursing students from four government universities located in the northern region of Saudi Arabia. Universities recruited in this study were labelled as University A, University B, University C and University D. Sample size calculations were undertaken using G*Power V.3.1 software (Faul et al. 2007) and a sample size of 252 participants across four universities was estimated to be adequate for this study.

### 3.2 Instrument

The Knowledge and Attitudes Survey Regarding Pain (KASRP) was developed in 1987 and updated in 2014 (Ferrell and McCaffery 2014). This scale allows students to self‐report their knowledge and attitudes related to pain and its management (Shdaifat et al. 2020). The KASRP is comprised of 39 items. The first 22 are true or false questions, followed by 15 multiple‐choice questions (items 23–37). The survey concludes with two case study questions (items 38–39). Scoring is based on the percentage of correct answers, with higher scores indicating a deeper knowledge and more positive attitudes towards pain management. Scores are divided into three categories: Poor (less than 50%), fair (50%–75%), and good (more than 75%) (Ferrell and McCaffery 2014). The reliability of the tool has been confirmed by the authors of the tool (Ferrell and McCaffery 2014), with a Cronbach's alpha of 0.70 and a test–retest reliability score of 0.80.

Concerns about the use of the KASRP tool—an English language tool—in the Saudi context have been raised in previous studies, specifically about the impact on participants' understanding of the items (Alsaqri 2018; Shdaifat et al. 2020). Therefore, the KASRP tool was translated into Arabic for the purpose of this study, with the approval of the original authors. This was undertaken following the five step approach described by Cheung et al. (2020). It included recruitment of the translation team, forward translation, backward translation, committee consolidation and pilot testing. The translation process resulted in minor modifications to some of the items to ensure relevance to the Saudi Arabian context. For example, in question (16) the generic name of the medication (Acetaminophen) was replaced by (Paracetamol), which is a more commonly used generic name in Saudi. In question 32, the word ‘United States’ was replaced by ‘Saudi Arabia’ to be more culturally relevant.

Content validity testing of the translated version of the tool was undertaken using Content Validity Index (CVI) as proposed by Yusoff (2019). The CVI is a four‐scale tool (1 = The item is not relevant to the measured domain; 2 = The item is somewhat relevant to the measured domain; 3 = The item is quite relevant to the measured domain; and 4 = The item is highly relevant to the measured domain). Ten nursing experts with expertise in pain management were recruited to validate the tool. An online survey including the Arabic version of the tool and the CVI scale was used to facilitate the process of validation. The results indicated a sound Scale‐Level Content Validity Index (S‐CVI/Ave) of 0.98 and Scale‐Level Content Validity Index, based on The Universal Agreement Method (S‐CVI/UA) of 0.85. According to Yusoff (2019), when recruiting at least nine experts, a CVI of 0.70 or above is considered acceptable. The reliability of the KASRP(A) (The Arabic translated KASRP tool) was established using SPSS software, and Cronbach's alpha was reported to be acceptable at 0.70.

### 3.3 Ethical Considerations

Prior to data collection, ethical approval number (ETH23‐8120) was sought and obtained from The University of Technology Sydney where the first author was enrolled as a doctoral student. Another ethical approval number (UT‐286‐129‐2023) was obtained from The University of Tabuk, one of the universities in Saudi Arabia under the ethical number (UT‐286‐129‐2023). These two approvals were sufficient to allow the authors to collect the data from the four governmental universities in Saudi Arabia. Potential participants were provided with a participant information statement which explained the study in detail and emphasised that participation was voluntary, the survey was anonymous, and consent would be implied by the submission of the survey.

### 3.4 Data Collection

Data collection was conducted between October 2023 and January 2024. Students who enrolled in their 3rd or 4th academic year of their bachelor's degree in nursing in the four selected universities were eligible to participate in the study. Students who were enrolled in any other academic year or in other universities other than the selected universities were not eligible to participate in the study. This sample was considered appropriate as the participants were senior students who had completed a number of core clinical placements and subjects covering pain management, meaning that they had the experience to meaningfully contribute to the study. The universities were purposively chosen from the same geographic region with comparable teaching facilities and cohort capacity to minimise institutional variation.

An advertising flyer inviting students to participate in the study was sent to the schools of nursing in the four targeted universities. The researcher also visited the schools, explained the aim of the study to potential participants, and answered any questions before distributing the link to the survey. Students who were willing to participate were asked to complete a survey in their own time using their smartphones or tablets, and they were again reassured that their participation was voluntary. The researcher was not present when the surveys were completed.

### 3.5 Data Analysis

Data were analysed using the RStudio Team (2020) data analysis software. Descriptive statistics were used to examine KASRP(A) scores (Ferrell and McCaffery 2014). Statistical significance was set at a *p*‐value of < 0.05. Differences between subgroups were examined using *t*‐tests. The original authors of the KASRP tool (Ferrell and McCaffery 2014) suggest not distinguishing between knowledge and attitudes items during analysis but to instead analyse the data as a percentage of the complete score. For the purpose of analysis, correct responses were given a score of (1) and incorrect responses were given a score of (0).

## 4 Results

### 4.1 Data Normality Assessment

To evaluate the distributional characteristics of the main continuous outcome variable, we employed both visual (Q‐Q plot) and statistical (Shapiro–Wilk test) methods. Results from the Shapiro–Wilk test indicated significant deviations from normality in larger subgroups, including students in the Regular Programme (*n* = 358, *p* < 0.001), 4th year students (*n* = 229, *p* < 0.001), and male students (*n* = 197, *p* = 0.018). In contrast, smaller subgroups such as those in the Bridging Programme (*n* = 35, *p* = 0.499) and students who had attended specific courses (*n* = 15, *p* = 0.414) did not show statistically significant departures from normality. However, these non‐significant results are likely attributable to the reduced statistical power associated with small sample sizes. Given the consistent evidence of non‐normality in the larger groups, the outcome variable was treated as non‐normally distributed in subsequent analyses.

### 4.2 Participant Characteristics

Three hundred and ninety‐three students responded to the survey from a population of 500 giving a response rate of 79%. The mean age of participants was 22 years (SD = 1.5), and the sample was almost equal in terms of gender. Most participants were in the 4th year of their nursing program (*n* = 229, 58%). More than 91% of participants reported that they had not worked in a healthcare setting prior to enrolling in a bachelor of nursing program. Only 4% reported that they had attended an extracurricular pain management course. The demographic characteristics of the total sample are reported in Table 1.

**TABLE 1:** Demographic characteristics for the total sample.

Demographic characteristics	*n* (393)	Percentage
Age (m = 21.6, SD = 1.5)		
< = 20 years	76	19.3
21–22 years	239	60.8
23–24 years	40	10.1
> = 25 years	38	9.6
Gender		
Male	197	50.1
Female	196	49.9
Educational program		
Bachelor of nursing	358	91.1
Bridging program^a^	35	8.9
Year of current enrolment		
3rd year	164	41.7
4th year	229	58.3
Educational Institution		
University A	185	47.1
University B	57	14.5
University C	83	21.1
University D	68	17.3
Prior experience of working in healthcare		
Yes	35	8.9
No	358	91.1
Setting for any previous healthcare work		
Surgical ward	6	1.5
Medical ward	5	1.3
Emergency room	3	0.8
Paediatric ward	0	0.0
Intensive care unit	4	1.0
Coronary care unit	1	0.3
Other	16	4.1
Context for learning about pain		
Clinical laboratories	25	4.3
Simulation unit	27	5.9
Clinical placements	81	32.6
All of the above	260	66.2
Attendance at an extracurricular pain management course		
Yes	15	3.8
No	378	96.2
Previous use of a pain scale		
Yes	329	83.7
No	64	16.2
Where the pain scale was used		
Clinical laboratories	17	4.3
Simulation unit	23	5.9
Clinical placements	128	32.6
All of the above	161	41.0
Frequency of using pain scale		
1–2 times	69	17.6
3–4 times	88	22.4
5–6 times	30	7.6
+6 times	142	36.1

^a^ Bridging program is program offered in Saudi universities for student with a diploma degree in nursing to get a bachelor's degree in nursing (usually bridging students have working experience).

### 4.3 KASRP Scores

The mean KASRP(A) score for the total sample in this study was 44% (SD = 8.8) indicating that, overall, the participants had low levels of knowledge and attitudes towards pain (see Figure 1). The percentage of correct responses for each question is shown in Table 2. Only 16 out of 41 items had a correct answer rate above 50%. Participants scored highest in the questions related to opioid management and safety in opioid administration and lowest in the case study questions related to pain management and methods for administering opioids in chronic conditions.

**FIGURE 1:**
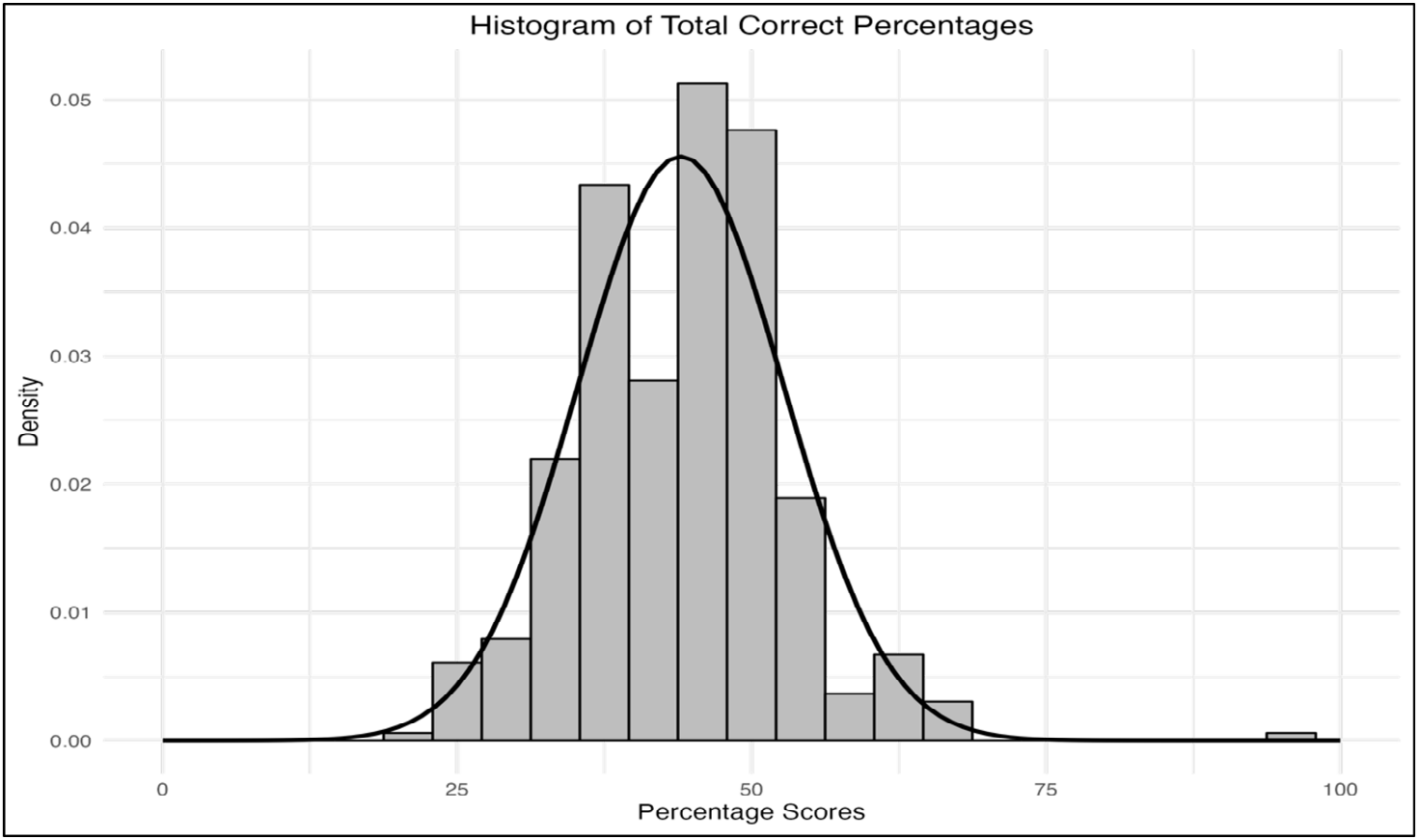
Histogram of the mean KASRP(A) score percentage of the study participant.

**TABLE 2:** Percentage of nursing students with correct responses for each question of the KASRP(A) (*N* = 393).

Items	*n*	Percentage
Q20 Drug/opioid analgesic addiction definition	363	92.4
Q14 Subsequent opioid doses should be adjusted according to the patient's own response	353	89.8
Q22 The necessity to assess the effect of the analgesic to avoid over‐analgesia and respiratory difficulty	349	88.8
Q21 The term ‘equianalgesia’ refers to the equivalence in the effect of analgesics that have a similar effect in relieving pain	336	85.5
Q13 Religious may influence the patient's belief that pain and suffering are necessary	331	84.2
Q16 Oral dose of Vicodin is approximately equal to 5–10 mg oral Morphine	273	69.5
Q34 The time it takes for intravenous Morphine to reach its maximum effect is	266	67.7
Q1 Vital signs can always be relied upon to assess a patient's pain intensity	241	61.3
Q6 Fixed doses of opioid analgesics over several months rarely suffer from respiratory depression	233	59.3
Q19 Benzodiazepines are ineffective in relieving pain and are rarely recommended when treating with analgesics	232	59
Q7 Taking a variety of analgesics that work in different ways, such as combining NSAIDs with opioids can improve pain with less side effects	228	58
Q29 The most likely reason a patient requests higher doses of analgesics is	228	58
Q25 Which of the following analgesics is considered the optimal choice for treating persistent moderate to severe pain in cancer patients?	221	56.2
Q24 The recommended method for administering opioid to patients with acute from physical injuries or post‐surgical pain, is	213	54.2
Q27 Initially, analgesics for pain relief after surgical operations should be taken	202	51.4
Q30 Which of the following medications is beneficial in treating cancer pain?	201	51.1
Q33 The likelihood of alcohol or drug use among patients suffering from pain is	196	49.9
Q2 Children under 2 years of age show less sensitivity to pain due to incomplete development of their nervous system, limited pain memory	179	45.5
Q12 Children under the age of 11 are unable to accurately express their pain, therefore doctors must rely entirely on the parents to assess the pain	177	45
Q10 Elderly patients cannot tolerate opioid analgesics for pain relief	175	44.5
Q32 Which of the following describes the best approach to considering cultural factors when caring for patients suffering from pain?	175	44.5
Q31 The person most capable of assessing the severity of a patient's pain is	174	44.3
Q5 Aspirin and nonsteroidal anti‐inflammatory drugs (NSAIDs) are not effective pain relievers for painful bone tumours	172	43.8
Q26 The equivalent dose for taking 30 mg of oral Morphine is	153	38.9
Q3 Patients who can be distracted from their pain usually do not suffer from severe pain	139	35.4
Q18 Taking anticonvulsants such as Gabapentin (Neurontin) effectively relieves pain after a single dose	134	34.1
Q4 A patient may be able to sleep despite severe pain	127	32.3
38A Case study 1A. Pain scale score in the absence of vital sign changes when the patients is relaxed	119	30.3
39A Case study 2A. Pain scale score in the absence of vital sign changes when the patient is grimacing	118	30
Q8 The duration of pain relief when taking a dose of 1–2 mg of morphine intravenously ranges from 4 to 5 h	101	25.7
Q9 The use of opioid analgesics should be avoided in patients with a history of drug abuse	101	25.7
Q11 Patients should be encouraged to endure pain as much as possible before taking opioid analgesics	98	24.9
Q37 The correct statement regarding respiratory difficulty caused by opioid analgesics is	98	24.9
Q36 After the sudden discontinuation of one of the opioid analgesics, physical dependence can manifest in the following symptoms	73	18.6
Q15 Administering sterile water by injection (placebo) to a patient is an effective test to determine whether the pain is real or not	63	16
Q28 The likelihood of the patient experiencing clinically evident respiratory distress during the increase of IV morphine dose	57	14.5
Q35 The time it takes for oral Morphine to reach its maximum effect is	50	12.7
Q17 When the source of pain is unknown, the use of opioid analgesics should be avoided during the evaluation period	49	12.5
Q23 The recommended method for administering opioid analgesics to patients suffering from persistent cancer pain is	40	10.2
38B Case study 2B. Administering morphine for pain as reported, without changes in vital signs or behaviours in a patient who shows grimacing	32	8.1
39B Case study 1B. Giving morphine for ongoing post‐surgery pain reported, with absence of vital signs or side effects, to a relaxed patient	18	4.6

#### 4.3.1 Opioid Management

Seventy‐five of the participants incorrectly believed that patients should be encouraged to tolerate pain as much as possible before taking opioids (Q11). In addition, 90% did not know that oral is the recommended method for morphine administration to individuals with cancer‐related pain (Q23). Moreover, 86% of participants incorrectly thought that patients are likely to experience respiratory distress after increasing the dose of morphine IV (Q28).

#### 4.3.2 Pain Assessment

With regards to the pain assessment item (Q29), 58% of participants reported that patients are more likely to request a higher dose of opioids due to increased pain; respectively, 19% and 14% of participants believed that patients are more likely to ask for more opioids due to addiction or anxiety (Q29); whereas 9% thought that patients request a higher dose to seek staff attention (Q29). In (Q31), less than half of the participants (44%) reported that the only reliable person to assess the intensity of the patients' pain is the patient themselves; whereas 26% and 19% thought that the nurse or physician is the only reliable person to assess the intensity of the patients' pain. Five percent of participants reported that the patient's spouse or family member is the only reliable person to assess the intensity of the patients' pain (Q31). More than 80% of participants mistakenly thought that use of a placebo is an effective way to assess pain (Q15).

The final part of the questionnaire consisted of two case study scenarios which were used to assess participants' decision‐making ability regarding pain assessment and intervention. The two case studies focused on patients of the same age who underwent the same surgical procedure and verbally reported the same level of pain based on the pain scale. In case study A, the patient is smiling and relaxed, while in case study B, the patient is grimacing and uncomfortable. The participants were asked to assess the patient's level of pain and the most appropriate nursing intervention. In response to both case studies (Q38A and Q39A), 30% of participants were able to correctly score the patient's pain. However, only 5% of participants thought it was appropriate to administer morphine 3 mg IV for the patient described in case study A, and 8% in case study B. Table 2 shows the number of correct responses for each item, ranked highest to lowest.

### 4.4 Differences in KASRP(A) Scores With Regards to Participants' Demographic Characteristics

Differences between KASRP(A) scores with regards to participants' demographic characteristics were examined with data clustered into two categories (Poor vs. Fair or Good). Among the 393 students, 308 (78.4%) had poor KASRP(A) scores and 85 (21.6%) had fair or good scores. The two groups were demographically similar: both were predominantly aged 21–22 years and enrolled in the regular program, with a slight male majority in the poor KASRP(A) score group (51.9%) and a slight female majority in the fair or good KASRP(A) score group (56.5%). Fourth‐year students made up a somewhat larger share of the fair or good group (62.4% vs. 57.1%). By institution, the poor KASRP(A) score group had a higher proportion from University A (49.0%), whereas the fair or good group had a higher proportion from University C (30.6%). The majority of students in both groups reported learning about pain across multiple contexts; however, this was more frequent in the fair/good KASRP(A) score group (72.9% vs. 64.3%). Prior use of a pain scale was high in both groups (87.1% vs. 82.8%), and frequent use of a pain scale (≥ 6 times) was somewhat more common among the fair or good KASRP(A) score group (40.0% vs. 35.1%). Table 3 shows the differences in demographics between subgroups.

**TABLE 3:** Differences in KASRP(A) scores (Poor vs. Fair and Good) with regards to participants' demographic characteristics.

Variables	KASRP(A) score (Poor)		KASRP(A) score (Fair and Good)	
	*N* = 308	Percentage	*N* = 85	Percentage
Age				
< = 20 Years	57	18.5	19	22.4
21–22 Years	185	60.1	54	63.5
23–24 Years	34	11.0	6	7.1
> = 25 Years	32	12.0	6	8.3
Gender				
Male	160	51.9	37	43.5
Female	148	48.1	48	56.5
Educational program				
Regular nursing program	278	90.3	80	94.1
Bridging nursing program	30	9.7	5	5.9
Year of enrolment				
3rd Year Nursing	132	42.9	32	37.6
4th Year Nursing	176	57.1	53	62.4
Educational institution				
University A	151	49.0	34	40.0
University B	47	15.3	10	11.8
University C	57	18.5	26	30.6
University D	53	17.2	15	17.6
Prior experience of working in healthcare				
Yes	30	9.7	5	5.9
No	278	90.3	80	94.1
Setting for any previous healthcare work				
Surgical ward	4	1.3	2	2.4
Medical ward	4	1.3	1	1.2
Emergency room	3	1.0	0	0.0
Paediatric ward	0	0.0	0	0.0
Intensive care unit	4	1.3	0	0.0
Coronary care unit	1	0.3	0	0.0
Other	14	4.5	2	2.4
Context for Learning About Pain				
Clinical labs	18	5.8	7	8.2
Simulation labs	25	8.1	2	2.4
Clinical rotation	67	21.8	14	16.5
All of the above	198	64.3	62	72.9
Attendance at extracurricular pain management course				
Yes	11	3.6	4	4.7
No	297	96.4	81	95.3
Previous use of a pain scale				
A. Yes	255	82.8	74	87.1
B. No	53	17.2	11	12.9
Where the pain scale was used				
Clinical labs	14	4.5	3	3.5
Simulation labs	22	7.1	1	1.2
Clinical rotation	100	32.5	28	32.9
All of the above	119	38.6	42	49.4
Frequency of using pain scale				
1–2 Times	56	18.2	13	15.3
3–4 Times	69	22.4	19	22.4
5–6 Times	22	7.1	8	9.4
+6 Times	108	35.1	34	40.0

Due to the non‐normality of the data, Wilcoxon rank‐sum (Mann–Whitney *U*) and Chi‐Square tests were used to examine relationships between KASRP(A) scores and demographic characteristics for the total sample (*n* = 393). Wilcoxon rank‐sum (Mann–Whitney *U*) test was applied when KASRP(A) scores were treated as continuous, while the Chi‐Square test was used when scores were categorised (Poor vs. Fair and Good). The result of the test (see Table 4) identified no significant differences between KASRP(A) scores and any of the demographic characteristics, including subgroup comparisons. For attendance at extracurricular pain management course variable, Fisher's exact test was used due to the small sample. However, no statistical difference was found.

**TABLE 4:** Relationships between KASRP(A) scores and demographic characteristics with two categories (*n* = 393).

Demographic characteristics	*n*	Mean (SD)	W. Statistic	*p*	Chi‐Square/Fisher's exact	*p*
Gender						
Male	197	17.7 (3.5)	17,562.0	0.11	1.57	0.21
Female	196	18.3 (3.6)				
Educational program						
Bachelor nursing program	358	18.0 (3.6)	6808.0	0.39	0.79	0.37
Bridging nursing program	35	17.5 (3.2)				
Year of current enrolment						
4th Year nursing	229	18.2 (3.7)	17,499.0	0.24	0.54	0.46
3rd Year nursing	164	17.7 (3.3)				
Prior experience of working in healthcare						
Yes	35	17.6 (3.2)	5852.5	0.51	0.79	0.37
No	358	18.0 (3.6)				
Attendance at extracurricular pain management course						
Yes	15	17.4 (3.7)	2719.0	0.78	N/A^a^	0.74
No	378	18.0 (3.5)				
Have you used pain scale						
Yes	329	18.0 (3.6)	10,997.0	0.57	0.60	0.43
No	64	17.7 (3.1)				

^a^ Fisher's exact test was used due to the small sample. N/A test result was reported as Fisher's test does not produce a test statistic.

For variables with more than two categories, two analytical approaches were taken to examine the relationship between the variables and the KASRP(A) score. Kruskal–Wallis tests were applied when KASRP(A) scores were treated as continuous, while the Chi‐squared/Fisher's exact were used when treating the data as categorical (Table 5). Educational Institution was the only variable showing a statistically significant relationship with KASRP(A) score when treated as a continuous variable (Kruskal–Wallis *p* = 0.01). This suggests that KASRP(A) scores significantly differ across institutions. When KASRP(A) scores were treated categorically (Poor, Fair and Good) the relationship with educational institution did not meet the conventional 0.05 threshold. Other variables including any previous healthcare work, context for learning about pain, where the pain scale was used and frequency of using pain scale did not demonstrate any statistically significant relationships with performance, under either continuous or categorical interpretations.

**TABLE 5:** Relationships between KASRP(A) scores and demographic characteristics with more than two categories (*n* = 393).

Variable	Kruskal–Wallis (Treated as continuous)		Chi‐squared/Fisher's exact (Treated as categorical)	
	Test statistic	*p*	Test statistic	*p*
Educational institution	0.33	0.01	6.33	0.09
Setting for any previous healthcare work	3.35	0.64	N/A^a^	0.70
Context for learning about pain	4.61	0.20	N/A	0.12
Where the pain scale was used	5.9	0.11	N/A	0.10
Frequency of using pain scale	3.59	0.30	1.04	0.79

^a^ Fisher's exact test was used due to the small sample. N/A test result was reported as Fisher's test does not produce a test statistic.

To further examine the significance of the KASRP(A) scores across universities, we employed two types of regression models. A logistic regression model was used to analyse binary categorical outcomes (i.e., Poor vs. Fair and Good), while a robust linear regression model (rlm) was applied to assess continuous outcomes. To account for potential heteroscedasticity, standard errors were adjusted using the HC1 estimator. This dual approach allowed us to evaluate the association from both categorical and continuous perspectives. Table 6 illustrates the two regression models comparison.

**TABLE 6:** Comparison of logistic and robust linear regression results.

Model type	Outcome variable	Term	Estimate	Std. error	Statistic	*p*
Logistic Regression	Performance category	(Intercept)	−1.491	0.19	−7.854	0.0
		University B	−0.057	0.397	−0.143	0.88
		University C	0.706	0.303	2.327	0.02
		University D	0.229	0.349	0.656	0.51
Robust Linear Regression	Total score	(Intercept)	17.399	0.259	67.05	0.0
		University B	1.208	0.417	2.895	0.004
		University C	1.227	0.464	2.644	0.008
		University D	0.573	0.499	1.149	0.25

The logistic regression model, used for binary categorical outcomes (Poor vs. Fair/Good), identified University C as statistically significant (*p* = 0.020), indicating higher odds of better performance compared to the reference university. In the robust linear regression model, which assessed continuous scores, both University B (*p* = 0.004) and University C (*p* = 0.008) showed significant positive associations with total scores, while University D did not reach statistical significance. These findings suggest consistent evidence of stronger performance by University C across both categorical and continuous models, while University B's association was significant only in the continuous outcome. Following a significant Kruskal–Wallis test and regression results, a Dunn post hoc test with Bonferroni correction was conducted to identify specific university pair differences in mean ranks, while controlling for family‐wise Type I error. The post hoc analysis indicated that University A's performance was significantly lower than that of both University B and University C. Figure 2 shows the result of Post hoc (Bonferroni) test result.

**FIGURE 2:**
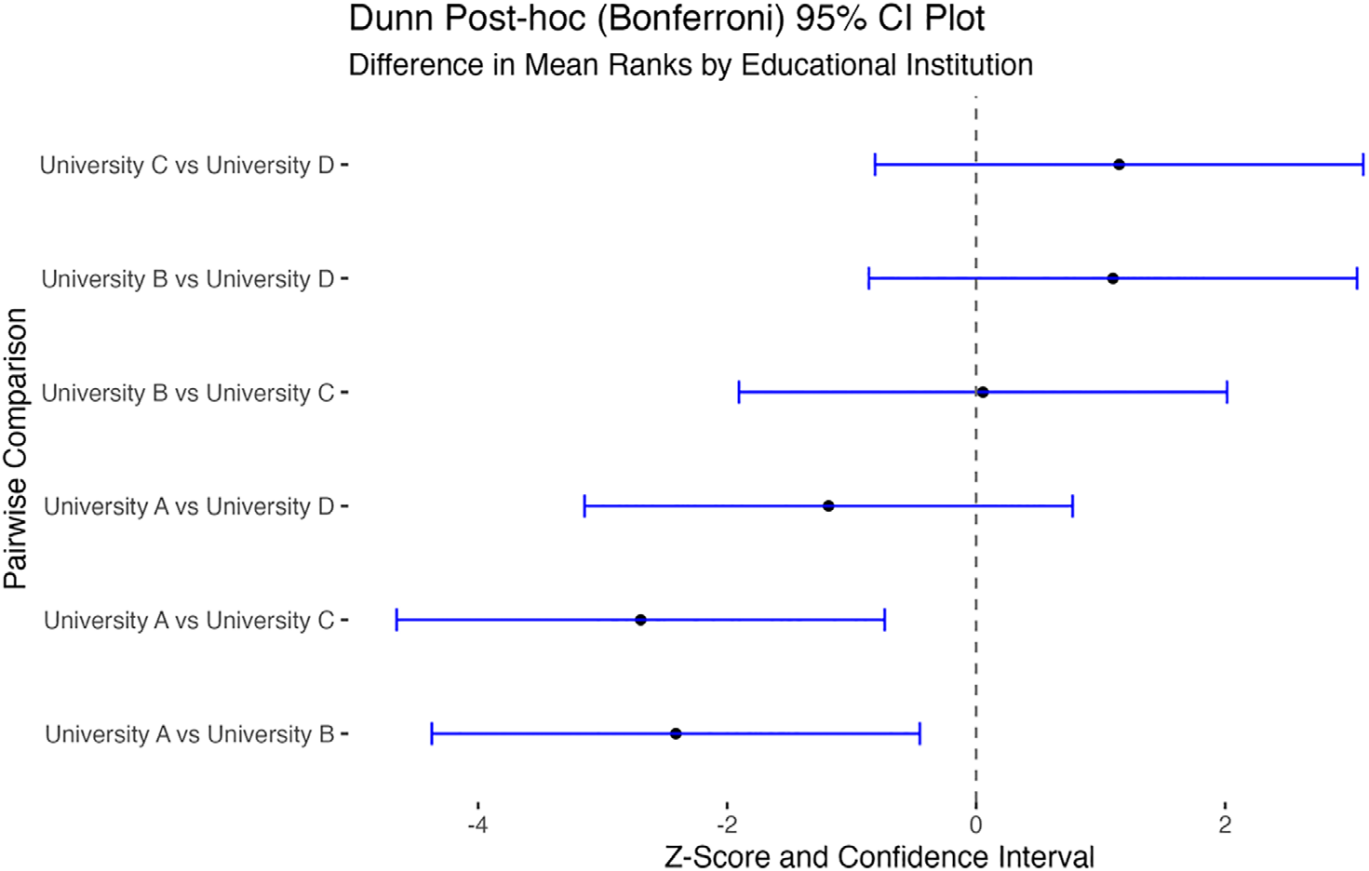
Dunn post hoc (Bonferroni) plot result.

## 5 Discussion

Quality nursing care requires patients to be provided with safe and effective pain management (Alruwaili et al. 2024). Educational institutions hold the responsibility for equipping future nurses with the knowledge, attitudes, critical thinking skills, and empathy required to deliver person‐centred care and effective pain management.

This study sought to examine Saudi nursing students' knowledge of and attitudes towards pain management. Overall, the results indicated that the participants' KASRP(A) scores were poor (< 50%) (McCaffrey and Ferrell 1997; Shdaifat et al. 2020). These results are consistent with a number of previous studies (Al Khalaileh and Al Qadire 2013; Al‐Khawaldeh et al. 2013; Alsaqri 2018; Gök and Topçu 2023; Hroch et al. 2019; Kusi Amponsah et al. 2020; Rababa et al. 2021; Shdaifat et al. 2020; Topal Hancer and Yilmaz 2020) and suggest that there is a need to explore how pain management is taught and assessed in nursing programs in Saudi universities (Alsaqri 2018; Shdaifat et al. 2020).

Previous studies recommended the translation of the KASRP to Arabic (Shdaifat et al. 2020; Wazqar 2019). After translating the tool for the purpose of this study, the average KASRP score of participants was still poor at 44% (SD = 8.8), although slightly higher than previous studies conducted in Saudi Arabia using the English language version of the instrument. For example, two studies were conducted in Saudi Arabia using the English language version of KASRP. A study by Alsaqri (2018) identified a mean score of 42% in the KASRP. In another study conducted in Saudi Arabia, the KASRP score was 43% (Shdaifat et al. 2020). It should be noted that the results of Saudi students in both the current and previous studies were not dissimilar to students from Jordan. For example, in a study by Al‐Khawaldeh et al. (2013) the mean KASRP score for Jordanian students was 34%, and in another study by Al Khalaileh and Al Qadire (2013) the mean score was 40%. It should be noted that other cohorts have achieved a ‘fair’ score (50%–75%). For example, a study of Canadian nursing students revealed a mean KASRP score of 67% (Hroch et al. 2019); and a similar US‐based study reported a mean KASRP score of 64% (Plaisance and Logan 2006).

To assess students' ability to engage in accurate decision‐making regarding pain assessment and the administration of opioids, the KASRP(A) includes two case study scenarios. In answering these questions, the majority of students were not able to identify the correct dose of analgesia to administer. This may indicate that Saudi nursing students receive limited education on pain assessment and opioid administration, a finding that aligns with the body of evidence on the topic (Al‐Khawaldeh et al. 2013; Rahimi‐Madiseh et al. 2010). Further, in the current study, participants demonstrated a lack of knowledge about aspects of pain management related to analgesic administration, including routes of administration, side effects of opioids and opioid tolerance. This finding is consistent with a previous study from the United States suggesting that some nursing students have a limited understanding of pain physiology and analgesic pharmacology (Duke et al. 2013). Lastly, the significant relationship identified between KASRP(A) scores and ‘educational institution’, may imply that differences in curricula, teaching methods, faculty, resources, or other institutional factors could impact students' knowledge, attitudes and perceptions related to pain management.

It is essential to ensure pain management education promotes future nurses' quality of care. The findings from this study identified a clear deficit in the participants' level of knowledge and attitudes towards pain management, indicating a need for curricula reform and greater attention to this critical issue (Erol Ursavaş and Karayurt 2021). Placing more emphasis on pain management during simulation may enhance student learning about pain management in a safe and interactive environment. Simulation experiences can also help improve students' confidence in communicating with people in pain as well as with other members of the multiprofessional pain team (Usman et al. 2025). Greater attention to strengthening students' pharmacological knowledge related to analgesic medications and pain management may help to reduce misconceptions about opioid administration.

## 6 Limitations

It is important to consider potential limitations when interpreting the results of this study. For example, cross‐sectional designs can identify correlations between variables but do not identify causality. Thus, the interpretation of the findings should be considered with a degree of caution. Additionally, the results cannot be generalised to all Saudi nursing students, or more broadly, due to the study being conducted in only four universities. Lastly, the use of a self‐report tool such as the KASRP(A) can increase the risk of social desirability bias.

## 7 Recommendations for Future Studies

There are several implications and recommendations that can be drawn from this study. The results could be used as a baseline to support Saudi policy makers and educators to review curricula, resources and teaching approaches in relation to the provision of evidence‐based pain management. The results may also indicate a need to raise students' level of awareness of the importance of pain management (Rababa and Masha'al 2020). Future research is recommended to repeat the study with a broader cross‐section of nursing students and potentially practicing nurses.

## 8 Conclusion

This study sought to examine Saudi nursing students' knowledge of and attitudes towards pain management. The results are of concern, and future studies should meaningfully explore the factors that may contribute to students' low levels of knowledge and poor attitudes regarding pain management. To this end, further studies with larger sample sizes as well as in‐depth qualitative approaches are recommended.

Managing pain effectively is a crucial part of high‐quality care, and it requires both well‐developed clinical skills and an understanding of how pain affects people in many different ways. This makes it essential for educational institutions to prepare graduates with the knowledge, skills, and attitudes needed to assess and manage pain safely, effectively, and with a person‐centred approach.

## Author Contributions

**Fawaz Abdullah Alshehri:** conceptualization, investigation, writing – original draft, methodology, visualization, validation, formal analysis, data curation, resources, project administration. **Tracy Levett‐Jones:** conceptualization, methodology, writing – review and editing, supervision. **Jacqui Pich:** conceptualization, methodology, writing – review and editing, supervision. **Abdulrahman S. Alamri:** conceptualization, formal analysis, visualization, writing – review and editing, software, data curation.

## Funding

The authors have nothing to report.

## Disclosure

Quality Report: The Strengthening the Reporting of Observational Studies in Epidemiology (STROBE) Statement: Guidelines for reporting observational studies was used in this study.

## Conflicts of Interest

The authors declare no conflicts of interest.

## Data Availability

Research data are not shared.
